# Evaluation of Parasight All-in-One system for the automated enumeration of helminth ova in canine and feline feces

**DOI:** 10.1186/s13071-024-06351-0

**Published:** 2024-06-27

**Authors:** Timothy Graham Castle, Leah Britton, Britt Ripley, Elizabeth Ubelhor, Paul Slusarewicz

**Affiliations:** 1 Eastern Kentucky University, 225 Park Dr, Richmond, KY 40475 USA; 2 Parasight System Inc., Suite 2130, 1532 N. Limestone St., Lexington, KY 40505 USA; 3 Lexington Humane Society, 1600 Old Frankfort Pike, Lexington, KY 40504 USA

**Keywords:** Parasight, Mini-FLOTAC, Imagyst, FEC

## Abstract

**Background:**

Digital imaging combined with deep-learning-based computational image analysis is a growing area in medical diagnostics, including parasitology, where a number of automated analytical devices have been developed and are available for use in clinical practice.

**Methods:**

The performance of Parasight All-in-One (AIO), a second-generation device, was evaluated by comparing it to a well-accepted research method (mini-FLOTAC) and to another commercially available test (Imagyst). Fifty-nine canine and feline infected fecal specimens were quantitatively analyzed by all three methods. Since some samples were positive for more than one parasite, the dataset consisted of 48 specimens positive for *Ancylostoma* spp., 13 for* Toxocara *spp. and 23 for *Trichuris *spp.

**Results:**

The magnitude of Parasight AIO counts correlated well with those of mini-FLOTAC but not with those of Imagyst. Parasight AIO counted approximately 3.5-fold more ova of *Ancylostoma* spp. and *Trichuris* spp. and 4.6-fold more ova of *Toxocara* spp. than the mini-FLOTAC, and counted 27.9-, 17.1- and 10.2-fold more of these same ova than Imagyst, respectively. These differences translated into differences between the test sensitivities at low egg count levels (< 50 eggs/g), with Parasight AIO > mini-FLOTAC > Imagyst. At higher egg counts Parasight AIO and mini-FLOTAC performed with comparable precision (which was significantly higher that than Imagyst), whereas at lower counts (> 30 eggs/g) Parasight was more precise than both mini-FLOTAC and Imagyst, while the latter two methods did not significantly differ from each other.

**Conclusions:**

In general, Parasight AIO analyses were both more precise and sensitive than mini-FLOTAC and Imagyst and quantitatively correlated well with mini-FLOTAC. While Parasight AIO produced lower raw counts in eggs-per-gram than mini-FLOTAC, these could be corrected using the data generated from these correlations.

**Graphical Abstract:**

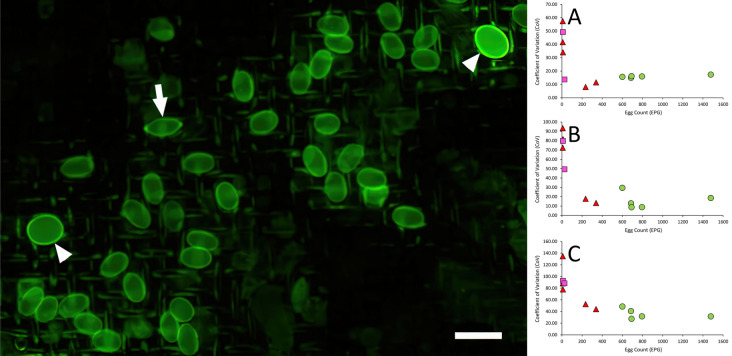

## Background

The diagnosis of infection by helminth parasites in veterinary practice is usually conducted by coproscopic examination of feces. Most commonly, fecal material is first suspended in a flotation medium (FM) where the specific gravity is high enough to allow parasitic diagnostic stages such as ova to float while the bulk of the fecal particulates sinks [1]. This separation facilitates the identification of ova among the otherwise overwhelming bulk of the remainder of the fecal sample. Over the years, a number of methods have been developed to exploit this general principle, including those that rely on either passive [2, 3] or centrifugal [4, 5] flotation, and many variants thereof [6]. In all of these cases, however, examination of the final sample is conducted manually by an analyst, which can lead to discrepancies arising from human error [7–9].

Modern technology has recently allowed this drawback of potential human error to be addressed through the use of advanced image processing and machine deep learning [10–12]. Two such methods are now commercially available for the diagnosis of helminth infection in domesticated cats and dogs (CADs). The first (Imagyst; Zoetis Inc., Kalamazoo, MI, USA) leverages deep learning to analyze high-resolution scans of samples taken at high magnification to automate the identification of ova [13, 14]. The second (Parasight System; Parasight System Inc., Lexington, KY, USA) adopts a different approach in that the system first chemically treats ova to expose their eggshells by oxidizing away the vitelline layer and then labels them with a fluorescently labeled chitin-binding protein [15]. The specificity and enhanced contrast facilitated by the fluorescence imaging modality facilitates image capture and analysis at a much lower magnification, leading to more rapid collection and analysis of the image data.

The Parasight System was originally developed solely to use with pasture animals [16–20]; its optical column was sufficient for this task, but not for the reliable detection/discrimination of some CAD ova (specifically *Ancylostoma* spp. and *Trichuris* spp.). The aim of the present study was to assess the performance of Parasight All-in-One (Parasight AIO), a second-generation device with enhanced optical capabilities and a radically different sample preparation methodology. To this end, we compared the Parasight AIO system to the mini-FLOTAC, a manual method commonly used by research parasitologists [2, 21–23], and to Imagyst, an alternative automated CAD fecal analysis system [13, 14].

When designing this study, we bore in mind the recent and concerning evolution of anthelmintic drug resistance in canine hookworms in the USA and Canada [24–29]. The ubiquitous nature of such resistance in equines and agricultural animals has led to the development of guidelines to aid in its detection using pretreatment quantitative fecal egg counts (FECs) and subsequent fecal egg count reduction tests (FECRTs) to monitor shedding reduction [30]. As a result, the American Association of Veterinary Parasitologists (AAVP) is now both recommending that veterinarians begin conducting both FECs and FECRTs in cases of *Ancylostoma canium* infection since drug efficacy can no longer be assumed and developing specific guidelines to address this issue [31].

With these developments in mind, in this study we paid particular attention to the quantitative aspects of Parasight AIO’s performance.

## Methods

### Samples

Samples were obtained from the Humane Society “Spay’s the Way” clinic (Lexington, KY, USA) and from the Veterinary Diagnostic Laboratory of Texas A&M University (College Station, TX, USA). The latter samples were shipped overnight on ice, and all samples were stored at 4 °C until analyzed. All analyses by all three methods were conducted within 1 week of each other.

The sample set consisted of 59 individual specimens (12 feline and 47 canine). A number of specimens were double- or triple-infected, and as a result the set contained 48 specimens that were positive for *Ancylostoma* spp., 13 specimens positive for *Toxocara* spp. and 23 specimens positive for *Trichuris* spp. We measured one random *Ancylostoma* spp. ova from a Parasight image from each of the 48 positive specimens and obtained average (± standard deviation [SD]) lengths of 60.3 ± 3.8 µm and widths of 40.6 ± 2.8 µm, suggesting that the ova were indeed members of the genus *Ancylostoma* and not *Uncinaria.*

### Analytical Methods

Manual analyses using the mini-FLOTAC method were conducted as described previously [2]. Slides were read using Nikon Eclipse E200 visible-light microscopes (Nikon Corp., Tokyo, Japan) at a magnification of 100×.

Imagyst analyses were conducted at two veterinary practices equipped with Imagyst devices and whose analysts had recently been trained by the manufacturer.

Parasight AIO analyses were performed as follows. Samples (1 g) were placed in 15-ml centrifuge tubes containing a single 9.5-mm ceramic ball and 9 ml of FM (specific gravity: 1.18 g/ml). After the tube was capped, samples were homogenized by vigorous shaking and then centrifuged at 2000 *g* for 1 min in a CF-800-1 fixed-angle centrifuge (Hardware Factory Store Inc., Azusa, CA, USA).

**Fig. 1:**
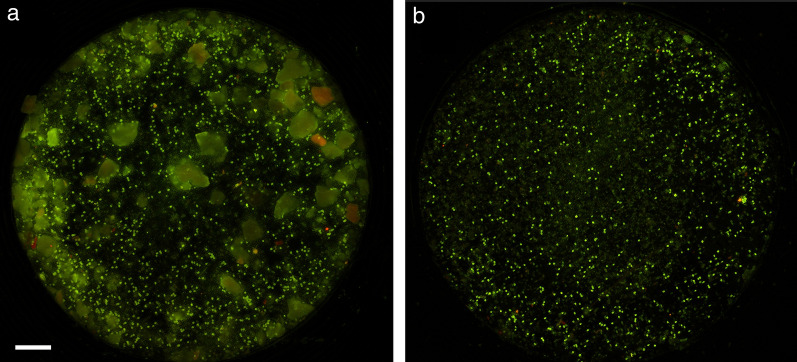
Effect of the egg separator tool on Parasight AIO image quality showing full Parasight AIO images from the same fecal sample processed either without (**a**) or with (**b**) the egg separator tool. Scale bar: 1 mm. AIO, All-in-One system

During initial development of the Parasight AIO system, it was noted that material in the post-centrifugal pellet could dislodge when the supernatant was being decanted and that this material could obscure some ova during subsequent analysis. To avoid this, we developed a tool (the egg separator [ES]) consisting of a hollow tube fitted at the distal end with a 130-µm mesh and an isoprene O-ring. When fully depressed into the 15-ml tube post-centrifugation, the O-ring produced a water-tight seal against the side of the tube while the mesh allowed the ova at the surface of the supernatant to pass through. In addition, the surface tension of the FM at the apertures of the mesh prevented any material below it (including that in the pellet) from escaping during decantation. This resulted in images that were substantially less contaminated with extraneous fecal material (Fig. 1).

**Fig. 2:**
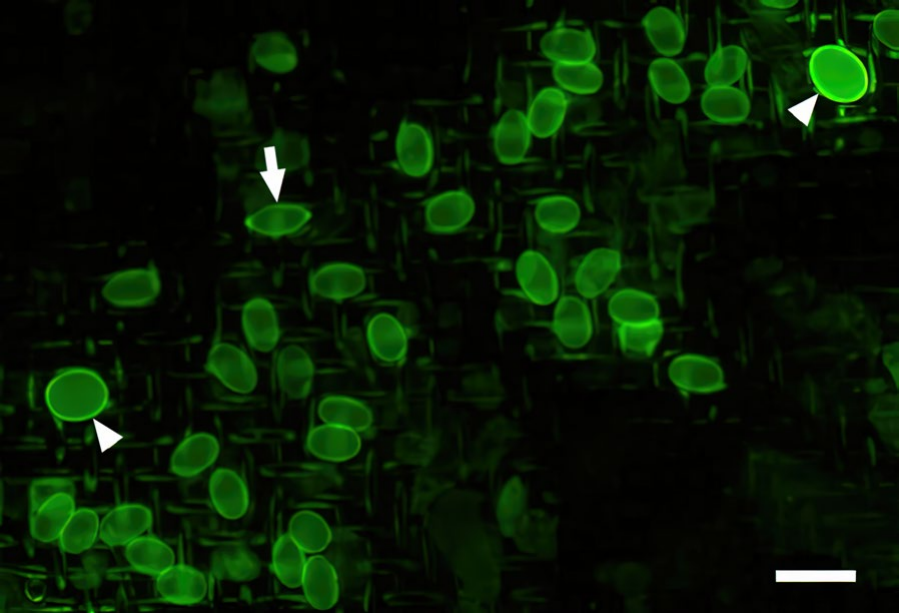
Parasight AIO example image. A portion of a Parasight AIO image containing two *Toxocara* spp. (arrowheads) and one *Trichuris* spp. (arrow) ova and multiple *Ancylostoma* spp. ova. Scale bar: 100 µm. AIO, All-in-One system

Thus, following centrifugation, an ES was pressed fully into the centrifuge tube and the supernatant decanted onto a fine mesh incorporated into a manifold termed an “Egg Chamber” (EC) that had been placed into its receptacle in the Parasight AIO device. After a vacuum drew the liquid through into the waste receptacle built into the EC manifold, the device proceeded to bleach, stain and wash the sample as described previously [15]. Once the chemistry had been completed, the device imaged the sample and performed an analysis using a deep-learning-trained algorithm to identify and enumerate any helminth ova present. A small representative portion of a Parasight AIO image is depicted in Fig. 2.

### Quantitative Analyses

The performances of the two automated methods (Parasight AIO and Imagyst) were compared to that of mini-FLOTAC, a widely accepted research method that has been the subject of a large number of validations and comparative studies [2, 21–23, 32–39].

Triplicate counts were generated from three independent subsamples of each fecal specimen for each analytical method (i.e. 9 total counts per sample, 3 per method). The means of the triplicate counts for each method were used for comparative purposes. While FECs can be over-dispersed, we chose to use means rather than medians due to the small sample size (*n* = 3); in such small datasets the possibility of two readings being outliers versus one is elevated, leading to a greater risk of discarding a “true” result and accepting an outlier.

### Precision

Method precision was assessed from the coefficients of variation (CoVs) determined from the repeated analysis (12 replicates) of the same samples by each method. Since this required large amounts of infected material, samples were generated by mixing infected material with a larger volume of non-infected material to produce ≥ 100 g of each sample. Homogenization was achieved by kneading each sample in a sealed plastic bag for 10 min, following which the sample was transferred to a plastic tub and mixed for a further 10 min with a spatula.

Five samples were generated in this manner from five different specimens that were positive for each of *Ancylostoma* spp. and *Trichuris* spp., respectively. Each sample was targeted to contain > 500 *Ancylostoma* spp. eggs/g. Two of the samples were triply infected and so also contained small numbers of *Toxocara* spp. ova.

Twelve independent 1-g subsamples of these specimens were analyzed using Parasight AIO and Imagyst, and 12 independent 5-g subsamples were analyzed using the mini-FLOTAC method.

### Statistical analysis

Calculation of Lin’s concordance correlation coefficients and comparisons of regression slopes were performed using the Real Statistics Resource Pack add-in (https://real-statistics.com/) for Microsoft Excel (Microsoft Corp., Redmond, WA, USA). The same package was used to calculate paired sample sign tests to compare egg count magnitudes between tests. CoVs were compared with Mann Whitney U-tests using JASP statistical software (v. 0.18.3; University of Amsterdam, Amsterdam, The Netherlands). Differences were considered to be significant at the *P* < 0.05 level.

## Results

**Fig. 3:**
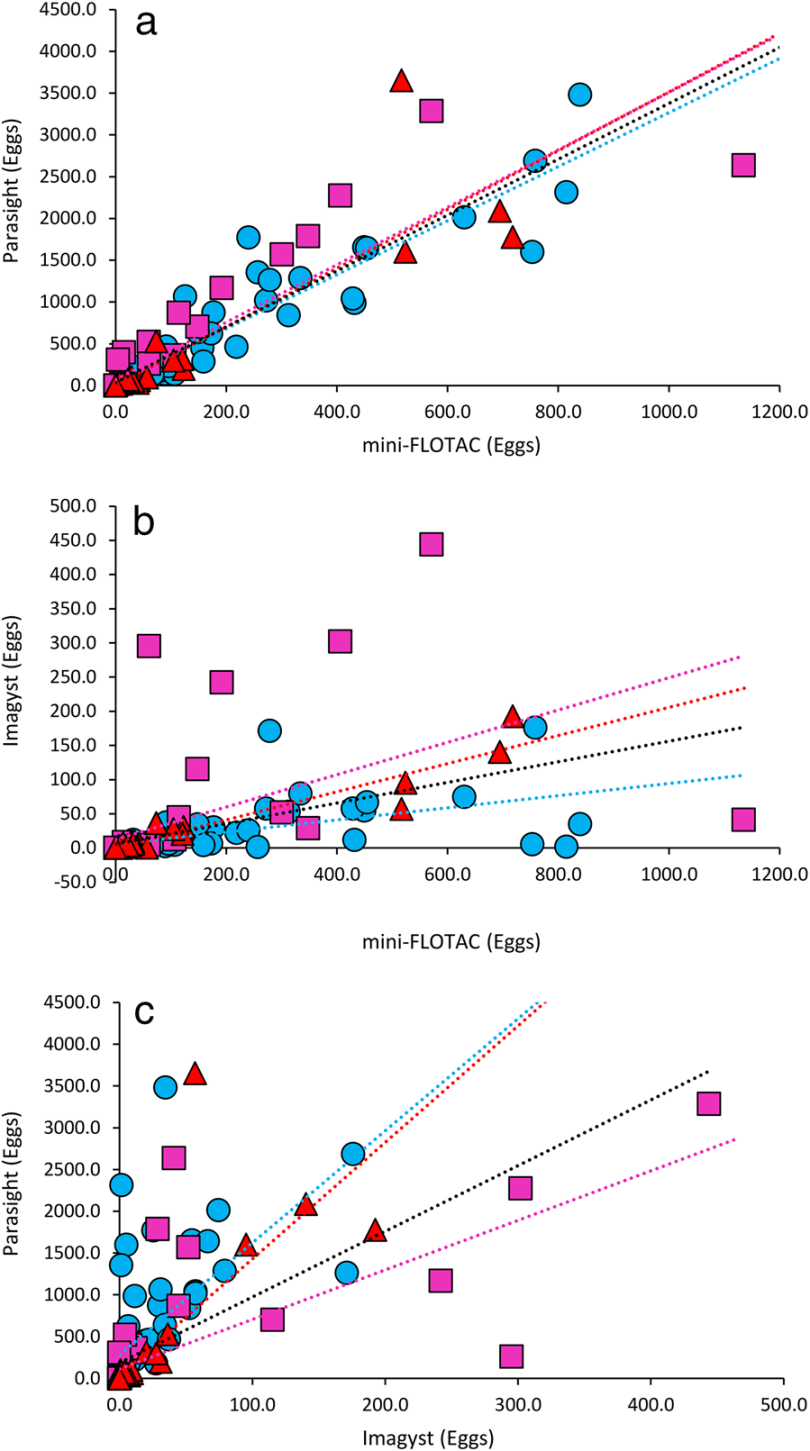
Correlation of counts generated by the three different methods. Correlation of Parasight AIO counts with mini-FLOTAC counts (**a**), of Imagyst counts with mini-FLOTAC counts (**b**) and of Parasight AIO counts with Imagyst counts (**c**). Blue circles indicate *Ancylostoma* spp.*,* magenta squares indicate *Toxocara* spp. and red triangles indicate *Trichuris* spp*.* The colored lines depict linear regressions for each egg type as well as for the entire dataset (black). AIO, All-in-One system

The number of eggs counted in each sample by mini-FLOTAC and Parasight AIO were well correlated and exhibited coefficients of determination of approximately 0.8 for each egg type (Fig. 3a; Table 1). While *Trichuris* spp. counts between mini-FLOTAC and Imagyst correlated strongly (*R*^2^ = 0.92), those for *Ancylostoma* spp. and *Toxocara* spp. did so poorly (Fig. 3b; Table 1). Correlations between counts by Parasight AIO and Imagyst were poor for all three egg types (Fig. 3c; Table 1).

**Table 1:** Summary of linear regression parameters for the comparisons of the three tests

Species tested	mFT vs. PS				mFT vs. Im				PS vs. Im			
	Slope	Intercept	*R*^2^	LinCCC	Slope	Intercept	*R*^2^	LinCCC	Slope	Intercept	*R*^2^	LinCCC
*Ancylostoma* spp.	3.2	34.3	0.88	0.41	0.09	5.6	0.31	0.13	13.3	287.8	0.38	0.04
*Toxocara* spp.	5.5	9.8	0.81	0.40	0.61	2	0.64	0.38	5.8	75.7	0.65	0.16
*Trichuris* spp.	3.5	− 1.4	0.8	0.41	0.21	− 0.1	0.92	0.37	13.9	35	0.58	0.08
All	3.7	21.2	0.84	0.42	0.18	4.1	0.31	0.25	7.8	175.4	0.41	0.08

Parameters are broken down for each egg type as well as for the entire dataset

*Im* Imagyst system, *LinCCC* Lin’s concordance correlation coefficient, *mFT* mini-FLOTAC method, *PS* Parasight All-in-One system

The slopes of the regression lines for both the three different egg species and the combined dataset were not significantly different when comparing Parasight AIO to mini-FLOTAC. In contrast, when comparing Parasight AIO to Imagyst, the slope of the *Toxocara* spp. regression line compared to those of both *Ancylostoma* spp. and *Trichuris* spp. was significantly different (*t*_(114)_ = 7.29, *P* < 0.0001 and *t*_(114)_ = 5.81, *P* < 0.0001, respectively), while the slopes of the *Trichuris* spp. and *Ancylostoma* spp. lines were not. In the case of mini-FLOTAC compared to Imagyst, there was no significant difference in the slopes of the *Toxocara* spp. and *Trichuris* spp. lines, but that of *Ancylostoma* spp. did differ significantly from those of *Trichuris* spp. and *Toxocara* spp. (*t*_(114)_ = 5.13, *P* < 0.0001 and *t*_(114)_ = 9.36, *P* < 0.0001, respectively).

The average number of eggs counted in each positive sample by each method and for each egg type was calculated (Table 2). In all cases, more ova were counted with the Parasight AIO system than with the mini-FLOTAC; in turn, more eggs were counted with the mini-FLOTAC than with Imagyst. These differences were all statistically significant for all three egg types and for the whole dataset (*P* < 0.0001). There were, however, differences in the magnitude of the count ratios depending on egg type. Parasight AIO counted 4.6-fold more *Toxocara* spp. ova than did the mini-FLOTAC but only approximately 3.5-fold more *Ancylostoma* spp. and *Trichuris* spp*.* When compared to Imagyst, Parasight AIO counted 27.9-, 10.2- and 17.1-fold more *Ancylostoma* spp.*, Toxocara* spp. and *Trichuris* spp. ova, respectively, while mini-FLOTAC counted 8.1-, 2.2- and 4.9-fold more.

**Table 2:** Mean absolute and relative egg counts for the three tests examined

Species tested	Mean eggs counted			Egg count ratios		
	mFT	PS	Im	mF/Im	PS/mFT	PS/Im
*Ancylostoma* spp.	205.4	708.2	25.4	8.1	3.4	27.9
*Toxocara* spp.	248.3	1154.5	113.6	2.2	4.6	10.2
*Trichuris* spp.	139.8	487.7	28.5	4.9	3.5	17.1

*Im* Imagyst system, *mFT* mini-FLOTAC method, *PS* Parasight All-in-One system

**Fig. 4:**
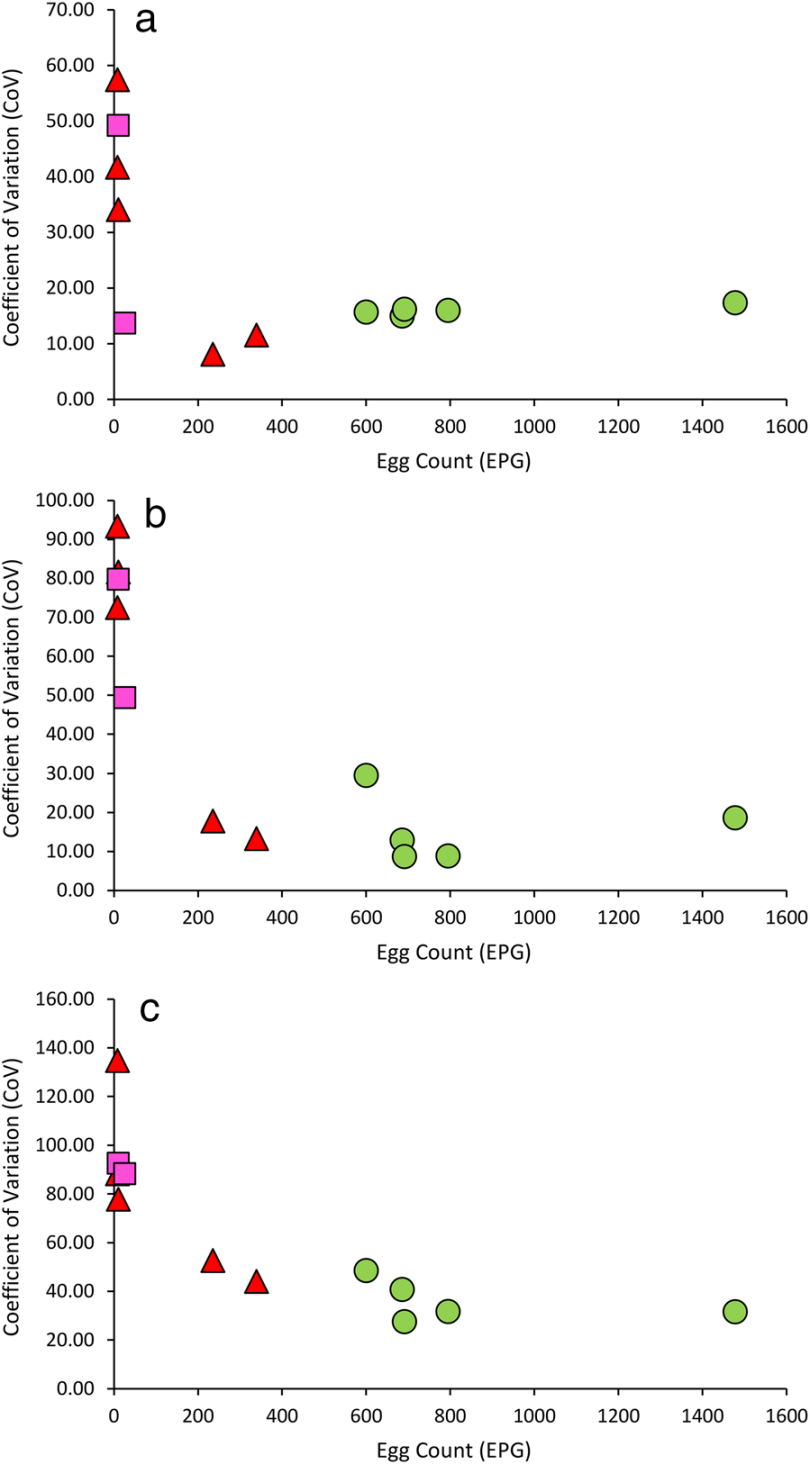
Precision of the three different methods. Coefficients of variation for Parasight AIO (**a**), mini-FLOTAC (**b**) and Imagyst (**c**) plotted against sample egg count as determined by mini-FLOTAC. Blue circles indicate *Ancylostoma* spp.*,* Magenta squares indicate *Toxocara* spp. and red triangles indicate *Trichuris* spp. EPG, Eggs per gram

The precision for all tests, as assessed using the CoVs of 12 replicate counts from five different fecal specimens, exhibited a strong dependance on the concentration of ova in the samples (Fig. 4). CoVs increased most dramatically at some point between 30 and 200 eggs per gram (EPG), at which point values remained relatively stable. CoVs from these two groups (low, containing 5 values, and high, containing 7 values) were analyzed separately (Table 3). There was no significant difference between the CoVs generated by Parasight AIO and mini-FLOTAC in the high group; however the CoVs for Imagyst were significantly higher than those obtained in the other two tests (*U* = 0, *Z* = 3.10, *P* = 0.0006, and *U* = 1, *Z* = 2.9, *P* = 0.0011 for Parasight and mini-FLOTAC, respectively). In the low group, however, the CoVs for Parasight AIO were significantly lower than those obtained by mini-FLOTAC (*U* = 1, *Z* = 2.30, *P* = 0.0159) and Imagyst (*U* = 0, *Z* = 2.51, *P* = 0.0079) while there was no significant difference between the CoVs for the mini-FLOTAC and Imagyst methods (*U* = 6, *Z* = 1.26, *P* = 0.22).

**Table 3:** Precision of the three tests examined in this study

Precision	Parasight AIO	mini-FLOTAC	Imagyst
High	14.3 ± 16.0	15.6 ± 36.2	39.5 ± 49.2
Low	39.3 ± 19.1	75.3 ± 30.4	96.4 ± 36.2

Numbers represent the mean (± standard deviation) coefficients of variation for samples in the low range (< 30 eggs per gram, *n* = 5) and the high range (*n* = 7)

*AIO* All-in-One

Both the quantitative and precision arms of the study afforded an opportunity to assess the sensitivity of all three tests. In this regard, samples from the quantitative arm of this study were classed as positive for any given parasite if at least one of the nine counts made (3 tests with 3 counts/test = 9 counts) contained ≥ 1 ova, while those in the precision arm were considered positive in any one of the 12 replicate counts for a sample that was positive. Thus, any negative counts found in the repeated counts of these samples were considered to be false-negatives.

The majority of false-negative results were observed in samples containing ≤ 50 EPG (as determined by multiplying the average of the repeated mini-FLOTAC counts for any given sample by the multiplication factor for that test, i.e. 5). To avoid suppressing the false-negative rate by including samples with high egg counts, only the samples whose counts fell below this threshold of 50 EPG were included in the analysis (Table 4). At counts of < 50 EPG and across all egg types, the false-negative rates were 7.3% for Parasight AIO (sensitivity = 92.7%), 18.8% for mini-FLOTAC (sensitivity = 81.2%) and 49% for Imagyst (sensitivity = 51%).

**Table 4:** False positive rates for the three tests examined in this study

Species tested	Samples (*n*)	Parasight AIO (%)	Mini-FLOTAC (%)	Imagyst (%)
*Ancylostoma* spp.	18	7 (38.9)	10 (55.6)	15 (83.3)
*Toxocara* spp.	27	0 (0)	1 (3.7)	12 (44.4)
*Trichuris* spp.	51	0 (0)	7 (13.7)	20 (39.2)
All	96	7 (7.3)	18 (18.8)	47 (49.0)

Results are from all counts for samples below 50 EPG and show the number (*n*) of tests for each egg type as well as the combined dataset. Numbers before the parentheses are the number of negative counts and those in parentheses are the % of false-negatives for each category

In addition, while the Parasight AIO and mini-FLOTAC methods generated no false-positive results in samples that contained > 50 EPG (i.e. above the threshold), Imagyst produced 12 false-positives, of which five occurred in samples with counts between 51 and 100 EPG, three in samples with counts between 101 and 200 EPG, two in samples with counts between 201 and 300 EPG and two in samples containing > 600 EPG.

## Discussion

Coproscopy in pasture animals is a quantitative procedure in which ova in test specimens are enumerated to generate FECs [40]. Quantitation serves two purposes: firstly to monitor the extent of anthelmintic drug resistance (which has become ubiquitous) in the population via FECRTs in order to inform treatment decisions [30, 41]; and, secondly, to reduce the number of ova/larva in pastures by targeting treatment to high-shedding individuals while allowing sufficient non-resistant ova to be shed to dilute drug-resistance genes within the local parasite population [42].

In contrast, due to the zoonotic nature of their helminth parasites, the coproscopic diagnosis of helminth infection in CADs has traditionally been solely concerned with determining the presence or absence of ova, and so sensitivity and specificity parameters have been the primary concern with respect to test performance.

This situation is likely to change in the near future due to the discovery of significant anthelmintic drug resistance in hookworms in the USA [24, 25], and the AAVP is now recommending the use of FECRTs in *A. canium* infections, with more detailed guidelines to follow [31]. As a result, we felt it important to not only assess the sensitivity and specificity of the Parasight AIO system but also its quantitative performance relative to a widely accepted research parasitology method (mini-FLOTAC) and an alternative automated system (Imagyst).

FECs generated by Parasight AIO correlated strongly with those generated by mini-FLOTAC, but both Parasight AIO and mini-FLOTAC counts correlated poorly with those generated by Imagyst, with the exception of *Trichuris* spp. counts, which correlated very strongly with those generated by the mini-FLOTAC (Fig. 3 and Table 1). The generally poor correlation between both Parasight AIO and mini-FLOTAC counts with most Imagyst counts suggests a greater variability in the counts generated by Imagyst compared to the other two methods. The Imagyst algorithm exhibits moderate agreement when the same slides are counted computationally and by trained parasitologists [13, 14], suggesting that the large variability observed here most likely also stems from any number of steps in the Imagyst process that differ to those of Parasight AIO and mini-FLOTAC. For example, the fecal slurries used in the Imagyst method are substantially more concentrated than those used in Parasight AIO and mini-FLOTAC (3.3 vs 0.1 g/ml, respectively). In addition, in Imagyst the raw slurry is filtered through a fine mesh filter during centrifugation while, in contrast, a coarse filter is used in the mini-FLOTAC and filtration in Parasight AIO only occurs post-centrifugation after the bulk of the fecal material has been sedimented. It is possible, therefore, that different physical characteristics of different fecal specimens may lead to differential fouling of the Imagyst filter during centrifugation, leading to differential egg recovery from sample-to-sample.

Furthermore, while Parasight AIO quantitatively analyses the entire subsample, which is poured onto the capture filter prior to staining and analysis, and mini-FLOTAC samples a fixed amount of a uniformly suspended slurry, Imagyst requires harvesting of the ova from the surface of the sample tube post-centrifugation using a custom sample loop. It is possible that variation can also be introduced to this process by inconsistent transfer of the ova to the liquid films on the sample loop and from the sample loop to the slide. Further studies are required to determine whether either or both of these possibilities are responsible for variations or whether other factors might be involved. It is unclear, however, why Imagyst generated a good correlation with mini-FLOTAC for *Trichuris* spp. ova, but not for *Ancylostoma* spp. and *Toxocara* spp. Further work is needed to determine whether this was a result of serendipity in the present study or a real effect that might be due to some physical or physicochemical properties of *Trichuris* spp. ova that render them more uniformly extractable than the other egg types.

Variability was more formally assessed by determining CoVs from multiple (12) counts of the same fecal samples by all three methods. While in this study we focused on *Ancylostoma* spp.*-*positive samples with moderate egg load due to these samples being more readily available, some were doubly or triply infected and contained low levels of *Trichuris* spp. and *Toxocara* spp. ova. This allowed us to also assess precision at low egg count levels. At these levels (< 30 EPG), CoVs for all tests were substantially higher (less precise) than in samples containing more eggs (Fig. 4). This finding was unsurprising because fecal subsampling is a Poisson process [43, 44]; in a Poisson distribution the mean equals the variance, and so populations with higher means have relatively lower CoVs (because CoV = √µ/µ, and √µ grows more slowly than µ with increasing µ).

At higher egg counts Parasight AIO and mini-FLOTAC produced very similar CoVs (mean: approx. 15) that were not significantly different, while both were significantly lower (more precise) than that of Imagyst (mean: 39.5), indicating lower precision of the latter method (Table 3). At lower egg counts, however, there was no significant difference in CoVs between the mini-FLOTAC and Imagyst CoVs (approx. 75 and approx. 96, respectively), while the CoVs of Parasight AIO (approx. 39) were significantly lower than both of the former methods. The lower precision displayed by Imagyst is consistent with the lower correlations observed between this method and the other two methods.

While the coefficients of determination for the correlations of all three egg types between Parasight and mini-FLOTAC were high, this was not the case for Lin’s concordance correlation coefficients (Table 1), which were substantially lower for all correlations between all methods. The reason for this was that the absolute numbers of eggs counted by each method were substantially different, leading to slopes that were substantially greater or smaller than unity.

Parasight AIO counted approximately 3.5- to 4.5-fold more ova compared to the mini-FLOTAC, depending on egg type, and 10- to 28-fold more ova compared to Imagyst; mini-FLOTAC counted approximately two- to eight-fold more ova than Imagyst (Table 2). The relative numbers of eggs counted by the methods varied between parasite species although the reason for the apparent differential yields is at present unclear and is worthy of further investigation. However, in all cases Parasight AIO counted more ova than mini-FLOTAC, which in turn counted more eggs than Imagyst.

In the case of Parasight AIO relative to mini-FLOTAC, the elevated count achieved by Parasight AIO is likely due to the sample size, with a full 1 of feces analyzed in the Parasight AIO system while the amount of material examined in a mini-FLOTAC cassette is the equivalent of 0.2 g feces. As a result, mini-FLOTAC counts are converted to EPG by using a multiplication factor of 5 (whereas since Parasight AIO and Imagyst both use 1 g of sample, no conversion is needed). Our results showing that Parasight AIO raw counts were 3.5- to 4.5-fold—and not fivefold—larger than those of mini-FLOTAC suggest a greater relative degree of egg loss during sample preparation in the Parasight AIO system. Possible sources of such egg loss include entrapment in the pellet during centrifugation, incomplete recovery of ova through the ES and obscurement of ova on the EC filter by fecal debris.

The counts for Imagyst were substantially lower (2-to 8-fold) than those for mini-FLOTAC—and not fivefold higher that would be expected based on mass alone. This suggests an even greater egg loss during sample preparation, perhaps for reasons such as those discussed above with respect to variability. This egg loss is further highlighted by the fact that even though Parasight AIO and Imagyst both use 1 g of feces for analysis, more eggs were counted by Parasight AIO than by Imagyst by more than one order of magnitude.

As a result of the observed undercounting of Parasight AIO and Imagyst relative to mini-FLOTAC, the latter represents the most accurate of the three tests because it produces the highest EPG values after the application of its multiplication factor. With that said, empirical data, such as those presented here, could be used to correct for these losses and produce “mini-FLOTAC equivalent” counts for both Parasight AIO and Imagyst after application of the appropriate experimentally determined correction factors [45].

Despite the higher egg loss, however, Parasight AIO’s ability to examine more feces in a single test still led to the detection of more ova per sample with this method than with mini-FLOTAC. Conversely, the large amount of egg loss exhibited by Imagyst led to the detection of fewer eggs per sample despite fivefold more feces being used in this method. This was, in turn, reflected in the relative sensitivities of the methods at egg counts of < 50 EPG (Table 4). Reflecting the order of count magnitude, Parasight AIO produced fewer false negative results than mini-FLOTAC, which in turn was more sensitive than Imagyst. At higher egg counts, both Parasight AIO and mini-FLOTAC produced no false negative results, but these were still produced occasionally by Imagyst at EPGs in the hundreds, and even in one sample in the thousands, possibly as a function not only of its lower egg yield but also some of the variability discussed above.

The findings in this study are broadly consistent with those of our previous evaluation of Parasight AIO in equines and its comparison to not only mini-FLOTAC and Imagyst but also to the McMaster and Wisconsin techniques [45]. In that study, Parasight AIO counted approximately similar numbers of eggs to the Wisconsin method, although further work would be required to determine whether this is also the case with CAD samples. Furthermore, mini-FLOTAC counted 2.7– to 5.6-fold more ova (strongyles and ascarids) than Imagyst, and Parasight AIO counted 2.8- to 3.4-fold and 9.1- to 15.6-fold more than mini-FLOTAC and Imagyst, respectively; these differences in count magnitudes had similar effects on the sensitivity of the tests, further demonstrating that tests that count more eggs are more likely to identify infection at low shedding levels.

It should be noted that 1-g samples are not always available in clinical practice, and so the use of rectal fecal loops, which extract only approximately 0.1 g of feces, is not uncommon [46]. A separate study to determine the relative sensitivities of the three tests evaluated here as well as those of other tests at these reduced sample volumes would therefore help to quantitatively illuminate the effect of this practice on the detection of parasitic infection.

The specificity of all three tests in this study was 100%, in that there were no false-positives detected. In cases where only one or two tests in a set of three triplicates (i.e. a total 9 counts of the same sample between the 3 methods) were positive with a small number of eggs, images from the positive (Parasight AIO or Imagyst) tests were examined to confirm a correct assignment. This would not be possible for mini-FLOTAC, since there is no photography associated with this method; however, there were no cases where mini-FLOTAC produced a positive result for a given sample where the other two tests were both negative.

The expectation is that manual methods such as mini-FLOTAC should operate with very high specificity when conducted by well-trained and diligent analysts, but this is not necessarily the case for computational algorithms. It should be noted that there were no negative samples in this study, since all samples were positive for at least one egg type. While 46 samples were negative for *Toxocara* spp. and 34 were negative for *Trichuris* spp., only 16 samples were negative for *Ancylostoma* spp., separate study utilizing negative samples would therefore be required to fully assess the specificity of these methods. Furthermore, while the present study could draw some conclusions about the relative accuracies of the compared methods, absolute determination of method accuracy can best be determined using spiking studies, where known numbers of ova are added to samples prior to analysis; such a study would be a worthy extension of the work presented here. In addition, assessment of clinical sensitivity was restricted to a relatively small number of low FEC samples and so a more extensive study using low-count naturally infected samples and/or samples spiked with low numbers of ova will be needed to in order to confirm the observations described here.

## Conclusions

The performance of Parasight AIO was equal to, or superior to, that of mini-FLOTAC with respect to precision, specificity and sensitivity, mainly due to the ability of the former to analyze more feces from a single sample and so detect more ova. The performance of Parasight AIO was equal to that of Imagyst with respect to specificity and superior in all other respects, even though both tests utilize the same amount of sample.

## Data Availability

All data generated or analyzed during this study are included in this published article.

## Abbreviations

AIO: Parasight All-in-One system
CAD: Cat and dog
CoV: Coefficient of variation
EPG: Eggs per gram
FEC: Fecal egg count
FECRT: Fecal egg count reduction test
